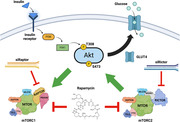# Elucidating the role of individual mTORC components on neuronal insulin signaling

**DOI:** 10.1002/alz.095339

**Published:** 2025-01-09

**Authors:** Jack Stahl, Aswathy Joji, Olga Khorkova, Claude‐Henry Volmar, Claes Wahlestedt

**Affiliations:** ^1^ University of Miami Miller School of Medicine, Center for Therapeutic Innovation, Miami, FL USA

## Abstract

**Background:**

Rapamycin is currently in clinical trials for AD, yet numerous studies have suggested that rapamycin inhibits mTORC2 as well as mTORC1, which could be detrimental for AD pathology. Brain insulin resistance is a known aspect of AD pathology and mTORC2 inhibition reduces AKT phosphorylation, which is a main mediator of cellular insulin signaling, perpetuating insulin resistance and further worsening brain glucose metabolism. Here, we show that rapamycin prevents insulin‐induced AKT phosphorylation in human neurons and explore the differential effects of mTORC1 and mTORC2 on neuronal insulin sensitivity.

**Method:**

Human neuronal cells were treated with 100nM rapamycin for 48 hours prior to collecting cellular protein for western blot analysis of S6 phosphorylation (mTORC1) and AKT phosphorylation (mTORC2). We then sought to specifically inhibit each complex by knocking down Raptor and Rictor, essential components for the formation of mTORC1 and mTORC2, respectively. Cells were transfected with siRNAs targeting Raptor or Rictor for 72 hours prior to collecting cellular protein for western blot analysis. For insulin experiments, cells were stimulated with 100nM insulin in serum‐free media for one hour prior to collecting protein for downstream analysis.

**Result:**

Treatment with 100nM rapamycin for 48 hours drastically decreased phosphorylation of ribosomal protein S6 (mTORC1) by 92% and phosphorylation of AKT (mTORC2) by 95%. While knocking down Rictor decreased mTORC2 activity by 71%, we were surprised to see that knocking down Raptor *increased* mTORC1 activity 2.1‐fold 72 hours post‐transfection. Treatment with 100nM rapamycin for 48 hours, which inhibits both mTORC1 and mTORC2, decreased total cellular protein and completely blocked insulin‐induced AKT phosphorylation at serine 473.

**Conclusion:**

Rapamycin has inhibitory effects on neuronal insulin signaling by inhibiting mTORC2, which can exacerbate AD pathology and symptoms. Our finding that knocking down Raptor increases mTORC1 activity contradicts the reduction in S6 phosphorylation seen with rapamycin treatment, a well‐established mTORC1 inhibitor, warranting further investigation into the role of individual mTORC components on neuronal metabolism. While mTORC1 inhibition has shown promise as a therapeutic strategy for AD, inhibition of mTORC2 can be detrimental for AD and elucidation of the metabolic role of each complex in the context of AD can guide future mTOR‐related therapeutic strategies.